# Multiomics Data Analysis Identified CpG Sites That Mediate the Impact of Smoking on Cardiometabolic Traits

**DOI:** 10.3390/epigenomes7030019

**Published:** 2023-08-22

**Authors:** Majid Nikpay

**Affiliations:** Omics and Biomedical Analysis Core Facility, Heart Institute, University of Ottawa, Ottawa, ON K1Y 4W7, Canada; mnikpay@ottawaheart.ca

**Keywords:** smoking, cardiometabolic traits, DNA methylation, Mendelian randomization, multiomics, pathway analysis

## Abstract

Understanding the epigenome paths through which smoking contributes to cardiometabolic traits is important for downstream applications. In this study, an SNP-based analytical pipeline was used to integrate several publicly available datasets in order to identify CpG sites that mediate the impact of smoking on cardiometabolic traits and to investigate the underlying molecular mechanisms. After applying stringent statistical criteria, 11 CpG sites were detected that showed significant association (*p* < 5 × 10^−8^) with cardiometabolic traits at both the discovery and replication stages. By integrating eQTL data, I found genes behind a number of these associations. cg05228408 was hypomethylated in smokers and contributed to higher blood pressure by lowering the expression of the *CLCN6* gene. cg08639339 was hypermethylated in smokers and lowered the metabolic rate by increasing the expression of *RAB29*; furthermore, I noted *TMEM120A* mediated the impact of smoking-cg17325771 on LDL, and *LTBP3* mediated the smoking-cg07029024 effect on heart rate. The pathway analysis identified processes through which the identified genes impact their traits. This study provides a list of CpG sites that mediates the impact of smoking on cardiometabolic traits and a framework to investigate the underlying molecular paths using publicly available data.

## 1. Introduction

Lifestyle choices could modify the risk of diseases partly through their impacts on the epigenome, which are genomic sites where the interaction of genetics and environmental factors occur. Regular tobacco smoking is known to impact a number of phenotypes, including cardiometabolic traits. The aim of this study was to investigate CpG methylation sites through which tobacco smoking impacts cardiometabolic traits. With the advancement of high-throughput screening methods, previous studies have identified CpG sites that show differential levels of methylation in smokers as compared to non-smokers. Genome-wide association studies (GWAS) also provided a comprehensive catalogue of biological entities (traits, biomarkers,…) and their underlying SNPs; meanwhile, analytical tools have been developed that can infer the relation between two entities using the knowledge available at the SNP level [1,2]. Motivated by these developments, in this study, an SNP-based analytical pipeline was devised to integrate the previous findings in order to investigate epigenome paths through which smoking contributes to cardiometabolic traits.

Findings from such a study could have different applications. First, many complex phenotypes, including cardiometabolic traits, progress gradually over time until they pass the liability threshold point and become diseases. As such, epigenomic biomarkers can greatly benefit preventive medicine because they allow health practitioners to detect the early presence of a disorder and monitor its condition over time. This is important because epigenomic changes are reversible by adjusting lifestyle. Second, at the molecular level, designing medications for every macromolecule (protein, metabolite, etc.) is not straightforward; however, with the development of a CRISPR-based epigenome editing system [3], targeting the epigenome sites underlying a trait could be a universal therapeutic solution applicable to various diseases. Finally, understanding the molecular path through which a lifestyle habit causes a disease is important for biological insight and downstream research.

The nature of the association between smoking, epigenome, and cardiometabolic traits has been the subject of a number of studies [4,5,6]. However, such studies normally measure DNA methylation and traits in the same group of subjects. Such a design does not differentiate between a confounding effect (habit ← epigenome change → disease), causation (habit → epigenome change → disease), or reverse causation (disease → epigenome change → habit). Furthermore, limited sample sizes hinder the power of such studies. Here, an analytical pipeline was used that relies on the concept of Mendelian randomization (MR) and allows the integration of data from large GWAS consortia. The approach is detailed in the Methods section.

## 2. Results

**Figure 1:**
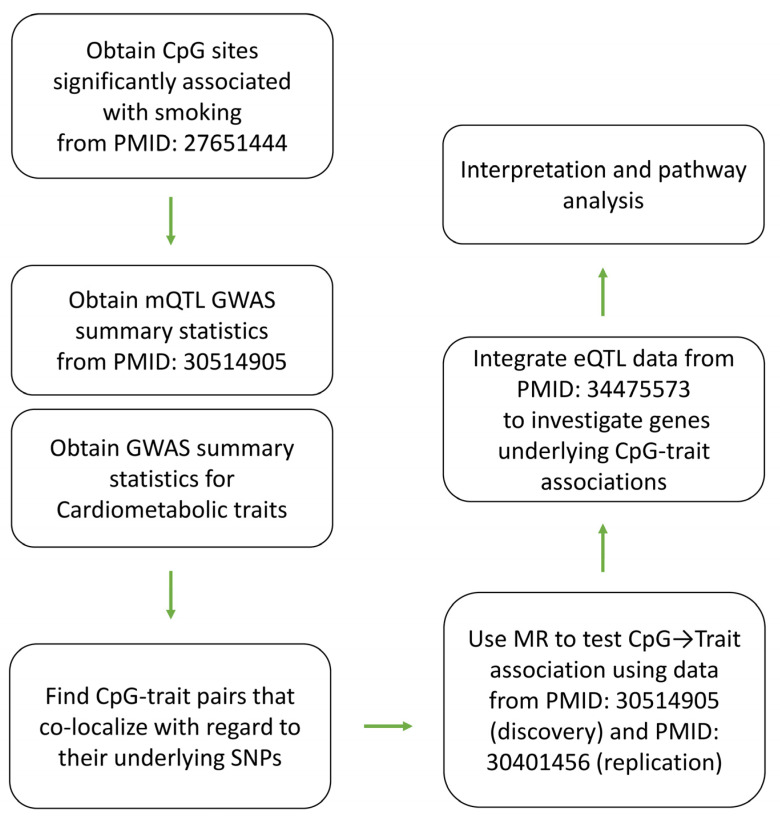
Overview of the analyses undertaken in this study to find CpG sites that mediate the impact of smoking on cardiometabolic traits. This study was conducted using publicly available data from previous studies. The list of CpG sites that showed differential levels of methylation between smokers and never-smokers (after the Bonferroni correction) was obtained from PMID: 27651444. Next, co-localization analysis was performed using GWAS summary statistics to identify candidate CpG-trait pairs for Mendelian randomization (MR). A two-stage discovery and replication design was used to identify CpG sites that significantly contribute to a cardiometabolic trait (*p* < 5 × 10^−8^). Next, eQTL data from the eQTLGen consortium were integrated to investigate genes that mediate the effect of a CpG site on a trait. Finally, pathway analysis was performed to investigate molecular mechanisms through which the identified genes impact their traits.

By choosing CpG sites that show differential levels of methylation between smokers and never-smokers in Joehanes et al.’s study [7], I examined their contribution to cardiometabolic traits using the SNP-based analytical pipeline described in Figure 1.

After applying rigorous statistical criteria, 11 CpG sites were identified that co-localized with cardiometabolic traits (Table S1) and significantly impacted their traits (*p* < 5 × 10^−8^) at both discovery and replication stages (Table 1 and Table S2).

The description of CpG sites and their nature of association with smoking is provided in Table S3. By inspecting data from the EWAS atlas [8], which is a repository of trait-epigenome modifications, I found confirmatory evidence from other studies with regard to the association of the identified CpG sites with smoking (Table S4). Next, eQTL data from the eQTLGen consortium [9] were integrated to investigate genes that mediated the impact of CpG sites on the traits. In the following sections, I review the notable findings:

### 2.1. Smoking Contributes to Hypertension by Hypomethylating the cg05228408 Site and Consequently Lowering the Expression of CLCN6

**Figure 2:**
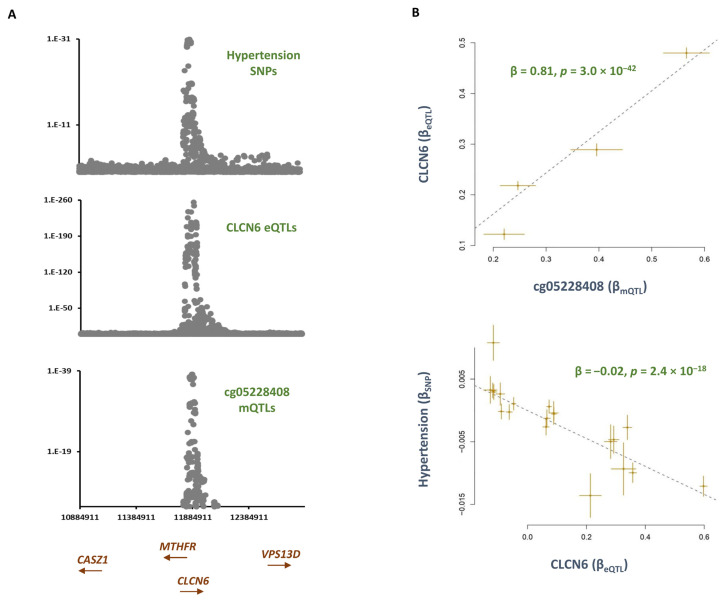
The mechanism whereby cg05228408 mediates the impact of smoking on hypertension. cg05228408 site is reported to be hypomethylated in smokers as compared to never-smokers (Tables S3 and S4). (**A**) Regional association plots for mQTLs of cg05228408, eQTLs of *CLCN6*, and risk SNPs of hypertension overlap. (**B**) MR analysis revealed as cg05228408 becomes hypermethylated, the expression of *CLCN6* increases, and this contributes to a lower risk of hypertension in non-smokers. Each point on the MR plots represents an SNP; the x-value of an SNP is its β effect size on the exposure, and the horizontal error bar represents the standard error around the β. The y-value of the SNP is its β effect size on the outcome, and the vertical error bar represents the standard error around its β. The dashed line represents the line of best fit (a line with the intercept of 0 and the slope of β from the MR test).

*AGTRAP-PLOD1* is a well-established locus for hypertension [10,11]. Within this locus, I found cg05228408 becomes hypomethylated in smokers as compared to never-smokers (B = −0.01, *p* = 6.4 × 10^−10^, Table S3) and consequently, this increases the risk of hypertension (Table 1). Several genes are located within the *AGTRAP-PLOD1* locus that are, to varying degrees, implicated in hypertension. By integrating the eQTL data, I noted GWAS signals for hypertension and cg05228408 overlap with eQTLs for *CLCN6* (Figure 2). The outcome of the MR analysis was also consistent; namely, higher methylation at the cg05228408 site was associated with higher levels of *CLCN6* (B = 0.81, *p* = 3.0 × 10^−42^, Figure 2), and consequently, this was associated with a lower risk of hypertension (B = −0.02, *p* = 2.4 × 10^−18^, Figure 2). *CLCN6* encodes a protein that acts as a voltage-dependent chloride channel; this protein is primarily localized to late endosomes and functions as a chloride/proton antiporter.

### 2.2. Smoking Increases the Methylation Level at cg08639339; This Lowers the Metabolic Rate by Increasing the Expression of RAB29

**Figure 3:**
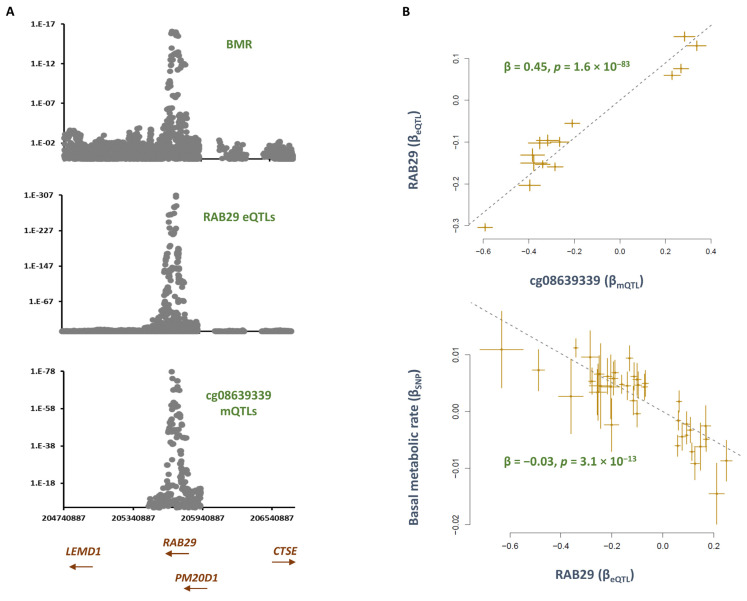
*RAB29* mediates the impact of smoking-cg08639339 on the basal metabolic rate (BMR) (**A**) I noted regional association plots for mQTLs of cg08639339, eQTLs of RAB29, and SNPs underlying the BMR overlap. (**B**) MR analysis revealed higher methylation at cg08639339 contributes to a higher expression of *RAB29*. This consequently contributed to a lower basal metabolic rate. Each point on the MR plots represents an SNP; the x-value of an SNP is its β effect size on the exposure, and the horizontal error bar represents the standard error around the β. The y-value of the SNP is its β effect size on the outcome, and the vertical error bar represents the standard error around its β. The dashed line represents the line of best fit (a line with the intercept of 0 and the slope of β from the MR test).

Co-localization analysis revealed mQTLs for cg08639339 overlap with SNPs, contributing to basal metabolic rate (P_SMR_ = 1.1 × 10^−11^, P_HEIDI_ = 0.07, Table S1). The top SNP in this region, rs6673687-T, was associated with a higher basal metabolic rate (B = 0.01, *p* = 3.2 × 10^−13^) but lower methylation at cg08639339 (B = −0.60, *p* = 1.5 × 10^−78^, Table S1). Consistently, the MR analysis revealed that higher methylation at this site contributes to a lower basal metabolic rate (B = −0.2, *p* = 3.6 × 10^−10^, Table 1). By investigating the eQTL data, I noted eQTLs for *RAB29* overlap with mQTLs for cg08639339 and GWAS signals for basal metabolic rate (BMR) (Figure 2). MR analysis revealed higher methylation at the cg08639339 site contributed to a higher expression of *RAB29* (B = 0.4, *p* = 1.6 × 10^−83^), and this lowered BMR (B = −0.03, *p* = 3.1 × 10^−13^, Figure 3). *RAB29*, formerly known as *RAB7L1*, encodes a protein that is involved in lysosomal trafficking and maintenance.

### 2.3. Smoking Contributes to LDL by Lowering the Methylation Level at cg17325771 and Consequently Enhancing the Expression of TMEM120A

**Figure 4:**
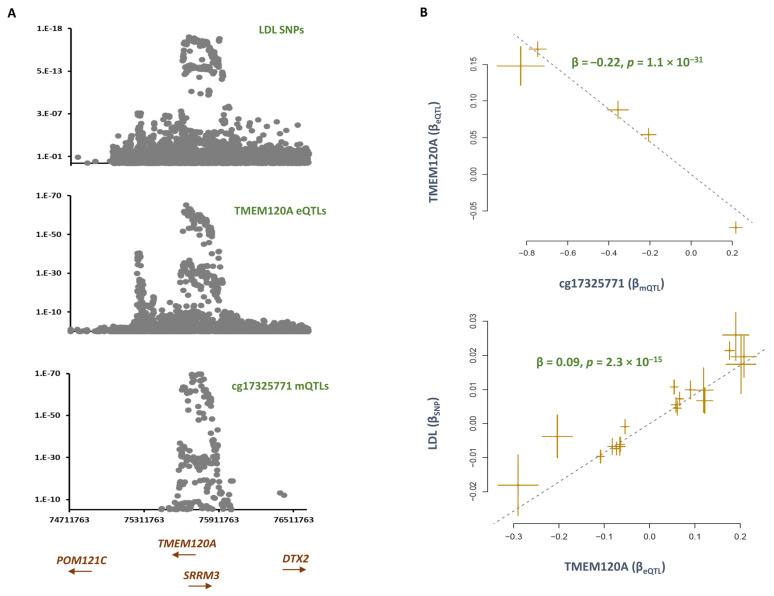
The mechanism whereby cg17325771 mediates the impact of smoking on heart rate. (**A**) Regional association plots for mQTLs of cg17325771, eQTLs of *TMEM120A*, and SNPs for LDL overlap. (**B**) MR analysis revealed that as cg17325771 becomes hypomethylated, the expression of *TMEM120A* increases, and this contributes to higher LDL. Each point on the MR plot represents an SNP; the x-value of an SNP is its β effect size on the exposure, and the horizontal error bar represents the standard error around the β. The y-value of the SNP is its β effect size on the outcome, and the vertical error bar represents the standard error around its β. The dashed line represents the line of best fit (a line with the intercept of 0 and the slope of β from the MR test).

The methylation site cg17325771 was hypomethylated (B = −0.01, *p* = 6.5 × 10^−11^) in smokers compared to non-smokers. Co-localization analysis revealed mQTLs for cg17325771 overlap with SNPs contributing to LDL (P_SMR_ = 3.4 × 10^−14^, P_HEIDI_ = 0.013, Table S1). Subsequently, MR analysis revealed lower methylation at this site contributes to higher LDL levels (B = −0.03, *p* = 6.9 × 10^−14^, Table 1). By plotting the distribution of eQTLs, I found *TMEM120A* to be the likely gene that mediates the impact of cg1732577 on LDL (Figure 4). The outcome of the MR analysis also revealed lower methylation at the cg17325771 site is associated with higher expression of *TMEM120A* (B = −0.22, *p* = 1.1 × 10^−31^), and this consequently contributes to higher LDL level (B = 0.09, *p* = 2.3 × 10^−15^, Figure 4). The protein encoded by *TMEM120A* is a transmembrane protein induced by the tumor necrosis factor-alpha (TNF-α).

### 2.4. Smoking Increases the Heart Rate by Increasing the Methylation Level at cg07029024 and Lowering the Expression of LTBP3

Among the identified CpG sites, cg07029024 showed the strongest association with smoking (B = 0.01, *p* = 5.5 × 10^−21^, Table S3). I noted mQTLs for this site, co-localized with SNPs impacting heart rate (P_SMR_ = 1.3 × 10^−8^, P_HEIDI_ = 0.07, Table S1). The MR analysis indicated higher methylation at this site is associated with a higher heart rate (B = 0.03, *p* = 1.5 × 10^−9^, Figure S1). The site is located on chromosome band 11q13.1. Among the genes in this region, I detected an association between cg07029024 and the expression of *LTBP3*. The outcome of analyses indicated as this site becomes methylated, the expression of *LTBP3* decreases (B = −0.7, *p* = 6.7 × 10^−21^), and this contributes to higher heart rate (B = −0.04, *p* = 3.4 × 10^−14^, Figure S1). *LTBP3*-encoded protein forms a complex with transforming growth factor beta (TGF-beta) proteins and may be involved in their subcellular localization.

### 2.5. From Genes to Pathways

To investigate the likely mechanism through which genes identified in this study impact their target traits, a pathway analysis was conducted by selecting pathways from the MSigDB database that contain these genes. Then, I examined whether SNPs that impact the activity of a pathway (measured by eQTLs associations) also impact the trait of interest (measured by SNP associations), by using the Spearman correlation test. Technical details of the analysis are provided in the Methods section. The results (Table 2) indicate that *RAB29* contributes to metabolic disorders through an immune path. The impact of *LTBP3* on heart rate is likely through processes involved in cell differentiation and proliferation. *TMEM120A* contributes to LDL through the PPARG-RXRA adipogenesis path. *CLCN6* contributes to blood pressure through the VEGEFA–endothelium path; moreover, the neural function of this gene may also have a role.

## 3. Discussion

Over the past two-decades high throughput studies have provided the research community with vast amounts of findings, which are continually being added to the databases. Currently, there are efforts toward joining these data for new purposes, and this study is another attempt in that direction. Lifestyle habits can predispose or protect us against diseases. At the molecular level, investigating the paths through which such changes happen is important for downstream applications such as early diagnosis, intervention, or to understand disease biology. The current study aimed to investigate the epigenome path through which smoking contributes to cardiometabolic traits using publicly available data.

Hypothetically, a significant association between a CpG site and a trait could represent a causal effect (CpG site → Trait), a reverse causal effect (Trait → CpG site), or a correlation because of the effect of unmeasured confounding factors (CpG site ← confounding factors → Trait). From the therapeutic perspective, the first scenario (the causal effect) is more important. A typical epidemiological study cannot differentiate between these scenarios. However, Mendelian randomization can control for both reverse causation and confounding because it uses a set of independent SNPs to judge the relation between two traits (please see the Methods section for more details). From the population perspective, the distribution of alleles of SNPs among individuals is unaffected by any environmental factor because alleles are randomly distributed at conception (according to Mendel’s laws). As such, if we divide a population sample into sub-samples according to the enrichment of risk alleles for a CpG site and compare the frequency of the disease between the sub-samples. We can judge whether a CpG site contributes to a disease or not.

Using a discovery and replication design and by setting stringent statistical criteria, I identified 11 CpG sites that mediated the impact of smoking on cardiometabolic traits. I found mQTLs for cg05228408, and eQTLs for *CLCN6* showed overlap with the GWAS signal for hypertension. The MR analysis further underlined this finding. I noted as this site becomes hypomethylated (as observed in smokers) the expression of *CLCN6* decreases, and this elevates blood pressure. The role of *CLCN6* in blood pressure regulation is known as discussed, GWAS and sequencing studies have linked mutations and variants within this gene to hypertension [10,11]. Recently, Klemens et al. provided functional evidence that *ClCN6* affects blood pressure by regulating Golgi calcium reserves, which in turn, contribute to vascular smooth muscle function [10]. The outcome of the pathway analysis conducted in this study was in line with this finding. Furthermore, it indicates the neural function of this gene could have a contribution. *CLCN6* is within the *AGTRAP–PLOD1* locus, which contains several genes implicated in blood pressure regulation, such as *MTHFR*, *NPPA*, and *NPPB*; therefore, as underlined earlier [11], further research is required to elucidate the role of this region in blood pressure regulation; however, finding from this study indicates hypermethylation of the cg05228408 site could represent a novel therapeutic intervention for lowering blood pressure. Furthermore, measuring methylation level at this site could represent a biomarker for early diagnosis of hypertension.

The analyses revealed that cg08639339 mediates the impact of smoking on basal metabolic rate through *RAB29*. A recent exome-sequencing study found this gene to be associated with cardiometabolic risk in the ARIC cohort [12]. The expression of *RAB29* is reported to be up-regulated in the presence of cholesterol biosynthesis [13]. *RAB29* encoded protein is involved in lysosomal trafficking and maintenance, and *RAB29* knock-out mice show lysosomal defects characterized by accumulation of lipids in kidney proximal tubule cells [14]. Pathway analysis underlined the role of this gene in metabolic disorder. Furthermore, it underlined immune paths as the likely mechanism through which this gene impacts metabolic rate.

*TMEM120A* is a trans-membrane protein that is known to be expressed in fat tissue and impacts adipogenesis/fat metabolism differentiation [15,16]. *TMEM120A* deficiency is reported to broadly impact lipid metabolism and causes lipodystrophy by altering genome topology [17]. Here, I identified a methylation site, cg17325771, within this gene that mediates the impact of smoking on LDL; furthermore, the outcome of pathway analysis indicated the contribution of this gene to LDL could be through the PPARG–RXRA adipogenesis path.

The cg07029024 site showed the strongest association with smoking. By integrating the eQTL data, I found *LTBP3* as the gene that mediates the impact of this site on heart rate. The outcome of pathway analysis indicated this gene likely impacts heart rate through processes involved in cellular development. *LTBP3* encodes latent TGF-β binding protein-3 (LTBP-3), which belongs to a family of proteins that regulate TGF-β activity by enabling its secretion, directing it to specific sites in the extracellular matrix, and participating in its activation. The role of LTBP3-TGF-β signaling in the differentiation of cardiac progenitor cells and formation of the heart has been researched [18]; besides, the *LTBP3* pathogenic variants are reported to predispose individuals to thoracic aortic aneurysms and aortic dissections [19]. Therefore, taken together, the impact of *LTBP3* on heart rate could stem from its role in cardiac maintenance.

In this study, 11 CpG sites were identified that mediate the impact of smoking on cardiometabolic traits; however, after integrating eQTL data, genes were identified for only 4 CpG sites. One reason could be due to the fact that CpG sites may act through non-coding elements. Therefore, it would be valuable if future studies extend QTL mapping to non-coding elements. This provides a more diverse set of data to investigate the mechanism through which a CpG site impacts a trait.

The analytical pipeline used in this study relies on publicly available data and can be applied to other lifestyle traits. This underlines the value of data sharing by researchers. In the long term, such practices will accelerate the functional annotation of the genome.

In this study, I took a conservative approach to lower the likelihood of false positives. Furthermore, mQTL data came from studies with relatively small sample sizes and a limited number of CpG sites. Future studies that integrate data from larger consortiums and dense methylation arrays are expected to provide a more comprehensive picture of epigenome sites that mediate the impact of smoking on cardiometabolic traits. In this regard, reporting trans-regulatory effects are very important because they appear to be common [20] but often remain unreported by the original QTL studies.

## 4. Conclusions

It is known that lifestyle habits modify the risk of diseases in part through their impact on the epigenome. A problem in this context is to identify the epigenome sites that mediate the impact of a lifestyle habit on a disease. This study describes a convenient framework to investigate the molecular path through which a lifestyle habit modifies the risk of a disease using publicly available data. By applying this framework to GWAS data for cardiometabolic traits, mQTLs, and differentially methylated CpG sites in smokers. A list of CpG sites was identified that mediates the impact of smoking on cardiometabolic traits. Furthermore, by integrating the eQTL and pathway data, the underlying molecular mechanisms were investigated.

## 5. Materials and Methods

### 5.1. Data Sources

Previously, Joehanes et al. [7] conducted a meta-analysis of genome-wide DNA methylation using DNA samples derived from the blood of 9389 participants (2433 current smokers and 6956 never smokers). The authors identified 2623 CpG sites that showed differential levels of methylation in current smokers vs. never-smokers after the Bonferroni correction. In this study, I chose these sites and examined their contribution to cardiometabolic traits through the analytical pipeline described in Figure 1.

### 5.2. Association with Cardiometabolic Traits

Initially, co-localization analysis was performed using the SMR program [1] to identify genomic regions where the GWAS signal for a CpG site and a trait overlap. The program can also differentiate between a pleiotropic effect (its null hypothesis) and a linkage effect using a test known as HEIDI. Following this stage, CpG-trait pairs that their underlying SNPs co-localize (P_SMR_ < 5 × 10^−8^) were selected. Next, Mendelian randomization was used to investigate CpG sites that are causally contributing to cardiometabolic traits.

Mendelian randomization (MR) is a statistical method that can investigate the nature of association between two biological entities by comparing their magnitude of associations to the same set of reference SNPs. Because the assortment of SNP alleles in offspring is a random process (unaffected by confounding environmental factors). Therefore, a set of SNPs can be used to investigate the nature of the association between two entities. In this study, I used the GSMR algorithm implemented in GCTA software [2] to conduct the MR analysis. SNPs that are used for MR analysis must pass a number of criteria to prevent bias in the assessment. First, they must not be in linkage disequilibrium. In this study, SNPs in which the extent of the LD among them does not exceed r^2^ < 0.2 (based on genotype data available from the 1000 genomes on subjects of European ancestry) were selected for MR analysis. Second, they must not show a pleiotropic effect (i.e., exposure ← SNP → outcome). Such SNPs were excluded from the instrument using the HEIDI test (*p* < 0.01) implemented in the GSMR algorithm. Third, they must be significantly associated with the exposure. For this purpose, SNPs that were associated with the exposure (CpG site) at GWAS significance level (*p* < 5 × 10^−8^) were selected.

mQTL summary statistics from McRae et al. [21] were used to conduct co-localization analysis and to find CpG sites that are causally contributing to a trait. The authors used the Infinium HumanMethylation 450 BeadChip (Illumina, San Diego, CA, USA) to measure DNA methylation in blood samples taken from 1980 subjects of European descent. GWAS summary statistics for cardiometabolic traits were obtained from the OpenGWAS database [22] by considering studies conducted using samples from the European population. This consideration is important to prevent bias due to population stratification.

CpG sites that were causally contributing to a trait (*p* < 5 × 10^−8^) and did not show evidence of reverse causation (*p* < 0.05) were selected, and their contributions were assessed once more using mQTL data from Hannon et al. [23,24]. Finally, I selected CpG-trait pairs with *p* < 5 × 10^−8^ and integrated eQTL data from the eQTLGen consortium [9] to identify genes that mediate the impact of methylation sites on traits.

### 5.3. Pathway Analysis

To systematically investigate the mechanism through which a gene may impact its trait. A pathway analysis was conducted by integrating GWAS, eQTL, and pathway data from MSigDB (v2023.1.Hs) [25,26]. To conduct the analysis, a list of pathways containing the gene of interest was obtained from the MsigDB. Next, for genes in a pathway, eQTL summary statistics were obtained from the eQTLGen consortium [9]. Then a pruning step was conducted (using PLINK 1.9 [27] clump function) to limit the list to eQTLs (*p* < 5 × 10^−8^) that are in linkage equilibrium (r^2^ < 0.05). Next, the correlation between |Z-scores| for eQTLs and GWAS SNPs was calculated (using Spearman’s rank correlation method) to test whether SNPs that impact a pathway also impact the trait of interest. The absolute values were used because genes in a pathway could have inhibitory or activatory functions.

## Figures and Tables

**Table 1:** CpG sites that mediated the impact of smoking on cardiometabolic traits.

CpG Site	Trait	Discovery			Replication		
		Beta	SE	*p*-Value	Beta	SE	*p*-Value
cg05228408	Hypertension	−0.03	0.003	2.3 × 10^−20^	−0.55	0.06	3.8 × 10^−23^
cg02998240	Low-density lipoprotein	−0.02	0.002	1.9 × 10^−21^	−0.29	0.03	2.2 × 10^−21^
cg01465596	Systolic blood pressure	−0.03	0.005	3.3 × 10^−11^	−0.53	0.08	6.0 × 10^−10^
cg08639339	Basal metabolic rate	−0.02	0.002	4.1 × 10^−11^	−0.32	0.05	1.5 × 10^−12^
cg27526649	Pulse rate	−0.48	0.06	4.8 × 10^−16^	−7.93	0.97	2.1 × 10^−16^
cg10676309	Basal metabolic rate	−0.03	0.005	8.2 × 10^−12^	−0.86	0.14	3.5 × 10^−10^
cg11105358	Immune reaction	−0.01	0.001	3.0 × 10^−10^	−0.21	0.03	3.6 × 10^−10^
cg05789250	Systolic blood pressure	−0.03	0.005	1.5 × 10^−9^	−0.82	0.15	1.7 × 10^−8^
cg12583553	Basal metabolic rate	−0.02	0.003	4.8 × 10^−9^	−0.31	0.05	5.7 × 10^−10^
cg12583553	Body fat percentage	−0.02	0.003	1.6 × 10^−10^	−0.32	0.05	2.1 × 10^−10^
cg17325771	Low-density lipoprotein	−0.03	0.004	6.9 × 10^−14^	−0.74	0.09	1.2 × 10^−15^
cg07029024	Pulse rate	0.03	0.004	1.5 × 10^−9^	0.39	0.06	3.0 × 10^−10^

The complete version of this Table is available as Table S2.

**Table 2:** Pathways that mediate the impact of genes identified in this study on their traits.

Trait	Gene Indicator	MSigDB ID	Description	r	*p*
Basal metabolic rate	RAB29	M1920	Gene network contributing to metabolic disorder	0.07	3 × 10^−14^
		M5017	Regulation of immune system	0.07	8 × 10^−14^
Heart rate	LTBP3	M4547	Regulation of cell differentiation	0.06	3 × 10^−10^
		M4627	Regulation of cell proliferation	0.06	6 × 10^−10^
LDL	TMEM120A	M2417	Genes targeted by PPARG and RXRA during adipogenesis	0.06	3 × 10^−8^
Hypertension	CLCN6	M2676	Genes up-regulated in endothelium by treatment with VEGFA	0.1	2 × 10^−5^
		M38335	Genes implicated in abnormality of central nervous system electrophysiology	0.06	6 × 10^−5^

## Data Availability

mQTL summary statistics were obtained from: https://yanglab.westlake.edu.cn/software/smr/#DataResource (accessed on 21 August 2023). eQTL summary statistics were obtained from the eQTLGen consortium: https://www.eqtlgen.org/ (accessed on 21 August 2023). GWAS summary statistics for cardiometabolic traits were obtained from: https://gwas.mrcieu.ac.uk/ (accessed on 21 August 2023). Gene sets in the MSigDB database were obtained from: https://www.gsea-msigdb.org/gsea/msigdb/human/collections.jsp (accessed on 21 August 2023).

## Supplementary Materials

The following supporting information can be downloaded at: https://www.mdpi.com/article/10.3390/epigenomes7030019/s1, Figure S1: The mechanism whereby cg07029024 site impacts heart rate; Table S1: The outcome of co-localization analysis between smoking-related CpG sites and cardiometabolic traits; Table S2: CpG sites that mediate the impact of smoking on cardiometabolic traits; Table S3: Association of CpG sites with smoking as reported in PMID: 27651444; Table S4: Confirmatory evidence for the association of CpG sites with smoking from the EWAS Atlas.
