# Supplementary material for: Multiomics Data Analysis Identified CpG Sites That Mediate the Impact of Smoking on Cardiometabolic Traits
Source: Epigenomes. 2023 Aug 22;7(3):19. doi: 10.3390/epigenomes7030019 (PMC10528714; doi:10.3390/epigenomes7030019)
Supplement: Supplementary file 1 [file epigenomes-07-00019-s001.zip › Figure S1.pdf]

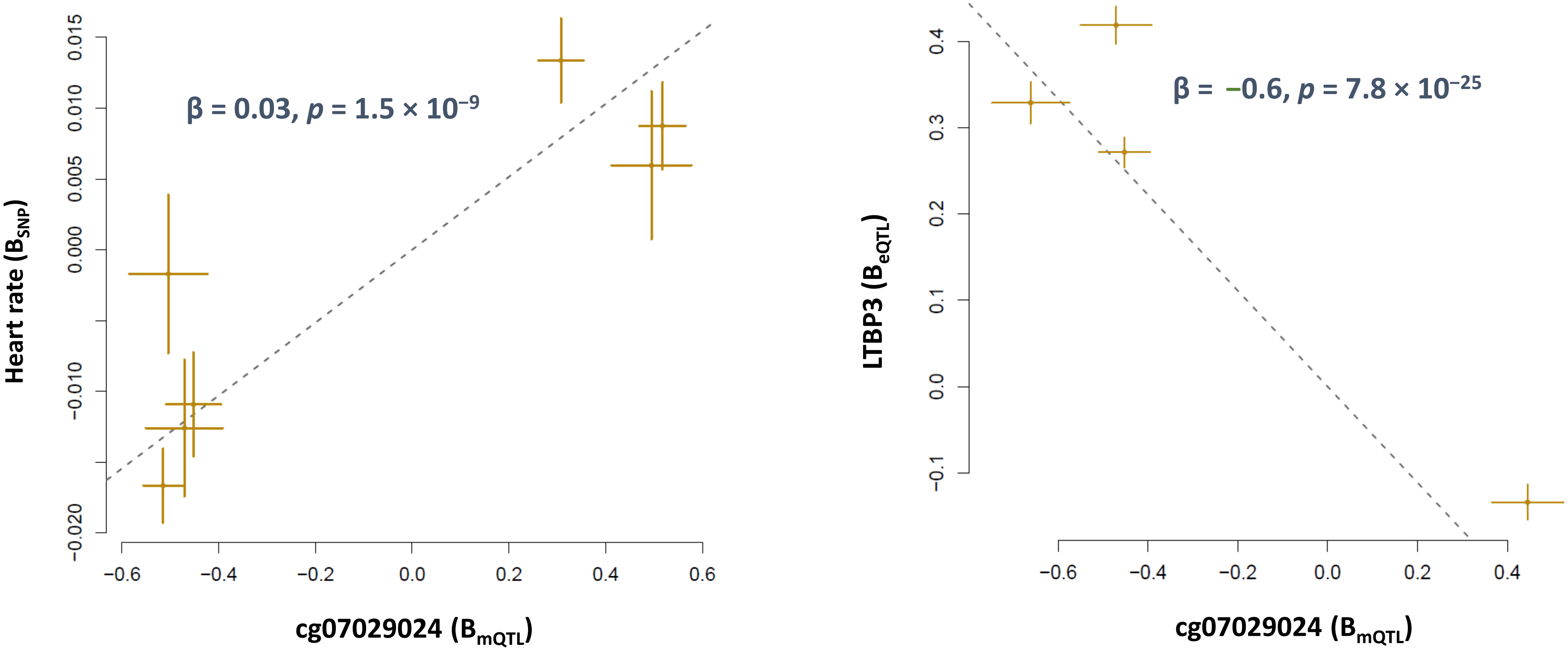

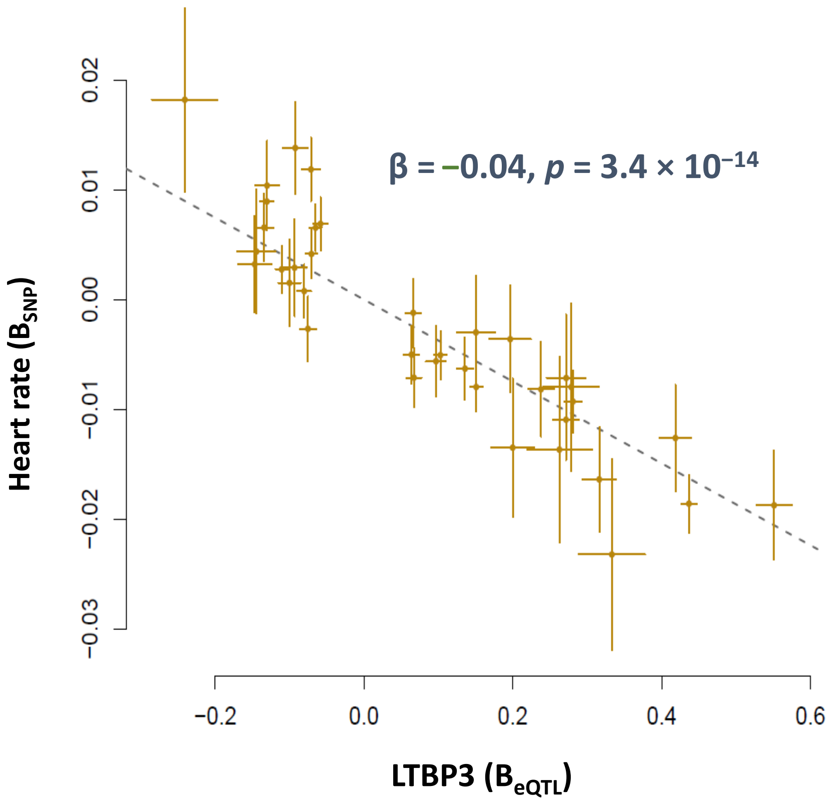

Figure S1. The mechanism whereby cg07029024 site impacts heart rate.
Higher methylation at cg07029024 (as a result of smoking) contributes to higher heart rate. By examining the eQTL data, I found *LTBP3* as the intermediary gene. Mendelian randomization revealed as cg07029024 site becomes methylated, the expression of *LTBP3* decreases and this consequently leads to higher heart rate. Each point on the scatter plots represents a SNP; the x-value of a SNP is its β effect size on the exposure, and the horizontal error bar represents the standard error around the β. The y-value of the SNP is its β effect size on the outcome, and the vertical error bar represents the standard error around its β. The dashed line represents the line of best fit (a line with the intercept of 0 and the slope of β from the MR test).
